# Changes in serum cytokines in response to musculoskeletal surgical trauma

**DOI:** 10.1186/1756-0500-7-128

**Published:** 2014-03-07

**Authors:** Olav Reikeras, Pål Borgen, Janne Elin Reseland, Staale Petter Lyngstadaas

**Affiliations:** 1 Department of Orthopaedics, Oslo University Clinic, Rikshospitalet, Oslo, Norway; 2 Martina Hansens Hospital, Gjettum, Norway; 3 Department of Biomaterials, Faculty of Dentistry, University of Oslo, Oslo, Norway

**Keywords:** Cytokines, Inflammation, Interleukins, Surgery, Trauma

## Abstract

**Background:**

Trauma induces local and subsequent systemic inflammatory reactions, and when the cytokine production is deregulated, a systemic inflammatory response syndrome with a potentially lethal outcome can occur. The understanding of the physiological mechanism of the cytokine network would be useful to better comprehend pathological conditions.

**Methods:**

We analysed a panel of 30 cytokines in the serum of 20 patients operated with total hip replacement. Cytokine release was assessed postoperatively up to 6 days by a multiplex antibody bead kit and compared to pre-operative values.

**Results:**

Surgery induced significant increments in serum levels of IL-2R at 6 days after surgery, in levels of IL-6 at 6 hours after surgery and at 1 day after surgery, in levels of IL-8 at 6 hours after surgery, in levels of IL-16 at 6 hours and at 1 day after surgery. Significant decreases in serum levels of IL-1Rα were found at the end of surgery, in levels of IL-12 at the end of surgery and at 6 hours after, and in levels of Eotaxin during all phases of the postoperative course.

**Conclusions:**

The major findings were significant increases in systemic levels of the pro-inflammatory cytokines IL-6, IL-8, IL-16, while IL-12 was significantly decreased. Otherwise there were modest changes in the systemic cytokine kinetics and no significant expression of anti-inflammatory cytokines.

## Background

Inflammatory cells that contribute to clearance and repair of necrotic tissue dominate the local response to injury [1]. These cells release soluble molecules, mainly cytokines, which generally function as intercellular messengers in an autocrine mode by binding to the cell of their origin or in a paracrine mode by binding to receptors on neighboring target cell [2].

Cytokines also act at sites distant from the origin of their production, and a systemic acute-phase response accompanies the local inflammation. This is followed by a compensatory anti-inflammatory response to attenuate the proinflammatory state [3], and the balance between the pro- and anti-inflammatory responses determines the net effect of an inflammatory response. In major injury disequilibrium between the pro- and anti-inflammatory responses may initiate a generalized response that in turn may progress to a multiple organ dysfunction [4].

Animal and human experiments have suggested the possibility of modifying the host inflammatory response, but clinical trials have been almost uniformly unsuccessful. The body’s response to trauma is a highly complex and heterogeneous sequence of events [5], and specific cytokine patterns, truly predictive of outcomes, are yet to be established. A difficulty has been in differentiating actual mediators of inflammation from inactive markers of inflammation. Thus, trauma models are required to provide a rational framework for the design of future clinical observational studies. We therefore sought to define the systemic release patterns of a broad panel of cytokines in a major standardized musculoskeletal trauma like total hip replacement.

## Methods

The study was approved by the Regional ethics committee and was performed in accordance with the ethical standards of the Declaration of Helsinki. After signed informed consent, 12 women and 8 men aged above 50 years that underwent primary cemented total hip arthroplasty (THA) due to osteoarthritis were included. All patients received spinal anesthesia without hypotensive effect with 5 mg/mL bupivacaine (Marcain®; AstraZeneca, Södertälje, Sweden) injected at the lumbar level.

We used thromboprophylaxis with low-molecular-weight heparin (dalteparin, ‘Fragmin’®; Pharmacia & Upjohn, Stockholm, Sweden) and infectious prophylaxis with cephfalothin (Keflin®; Eli Lilly, Indianapolis, IN., USA). Voluven® and Ringer’s acetate (Fresenius KABI, Bad Homburg, Germany) were used as plasma substitutes. The operation was performed in the lateral position, using a standardized posterior approach. Postoperative analgesia was administered according to a standard protocol consisting of paracetamol and codeine sulphate (Paralgin forte®; Weifa AS, Oslo, Norway) and ketobemidon (Ketorax®; Jenahexal Pharma, Jena, Germany). Closed postoperative drainage was used for 24 hours. All patients were mobilized on the first postoperative day.

Patients with allergy to dalteparin, bleeding disorders, renal failure, hepatic disease, active treatment for malignancy, on-going antithrombotic treatment, history of deep vein thrombosis or pulmonary embolus, and patients experiencing major operations, traumas, stroke, or cardiac infarction the last 3 months before surgery were excluded. Patients were advised to stop antiplatelet medication 1 week before surgery.

Hemoglobin, hematocrit, white blood counts, platelet counts, c-reactive-protein, creatinin, and liver enzymes were analyzed the day before surgery.

Blood samples were obtained from peripheral veins at the following time points: (T1) before induction of anesthesia, (T2) at the end of surgery, (T3) 6 hours after surgery, (T4) the day after surgery, and (T5) 6 days after surgery. Blood samples was kept on ice until it was separated by centrifugation at 2500 g for 20 min at 18 degrees C and stored at -80 degrees C until assayed. The concentration of cytokines in the blood samples was determined by a multiplex antibody bead (Chemokine/Cytokine 30-Plex, Biosource, Camarillo, CA, USA) and were simultaneously measured in the Luminex-100 system according to the manufacturer’s instructions. The acquired fluorescence data were analyzed by Starstation software (version 2.0; Applied Cytometry Systems, Sheffield, United Kingdom).

Statistical analyses were performed using SPSS II software Version 19 (IBM Inc. USA). Data are presented by mean and standard deviation. Time dependent changes were performed by analysis of variance (ANOVA). If significant differences were indicated, we used the LSD post hoc test. P ≤ 0.05 was considered significant. Correlations were carried out with Pearson correlation.

## Results

The operative time ranged from 44 to 119 minutes with a mean of 68 minutes, and the postoperative course was uneventful in all patients up to 6 days after surgery when they left the hospital. Surgery induced significant increments in serum levels of Interleukin-2 receptor (IL-2R) at 6 days after surgery (p = 0.014), in levels of IL-6 at 6 hours after surgery (p = 0.020) and at 1 day after surgery (p = 0.003), in levels of IL-8 at 6 hours after surgery (p = 0.006) and in levels of IL-16 at 6 hours after surgery (p = 0.019) and at 1 day after surgery (p = 0.002) (Table 1). Significant decreases in serum levels of IL-1 receptor alpha (IL-1Rα) were found at the end of surgery (p = 0.044), in levels of IL-12 at the end of surgery (p = 0.047) and at 6 hours after surgery (p = 0.018) and in levels of Eotaxin during all phases of the postoperative course (p = 0.018, 0.001, <0.001 and 0.005, respectively).

**Table 1:** Changes in serum cytokines (pg/mL)

	**T1**	**T2**	**T3**	**T4**	**T5**	**ANOVA**
Il-1Rα	441 ± 118	345 ± 125^a^	363 ± 191	398 ± 119	498 ± 189	0.011
Il-2R	231 ± 70	179 ± 66	187 ± 86	231 ± 67	346 ± 29^b^	0.003
Il-6	13 ± 33	14 ± 27	33 ± 23^c^	41 ± 29^d^	22 ± 26	0.008
Il-8	8.9 ± 4.3	10.0 ± 4.7	18.2 ± 22^e^	10.4 ± 4.7	15.1 ± 6.4	0.028
Il-12	135 ± 38	106 ± 37^f^	100 ± 46^g^	111 ± 36	141 ± 67	0.018
Il-16	0.3 ± 0.9	0.6 ± 0.8	1.0 ± 0.7^h^	1.2 ± 1.0^i^	0.7 ± 0.9	0.010
Eotaxin	66 ± 20	50 ± 25^j^	44 ± 26^k^	38 ± 18^l^	47 ± 16 ^m^	0.001

Time points are before induction of anaesthesia (T1), at the end of surgery (T2), at 6 hours after surgery (T3), at the day after surgery (T4), and at 6 days after surgery (T5). Values are mean ± standard deviation.

^a^p = 0.044, ^b^p = 0.014, ^c^p = 0.020, ^d^p = 0.003, ^e^p = 0.006, ^f^p = 0.047, ^g^p = 0.018, ^h^p = 0.019, ^i^p = 0.002, ^j^p = 0.018, ^k^p = 0.001 ^l^p = <0.001, ^m^p = 0.005 in relation to T1.

In the postoperative course up to 6 days there were no significant changes in serum levels of IL-1β, IL-2, IL-5, IL-7, IL-10, IL-13, IL-15, IL-17, Tumor necrosis factor alpha (TNF-α ), Interferon alpha (INF-α), Interferon gamma (INF-γ), Granulocyte macrophage colony-stimulating factor (GM-CSF), Macrophage inflammatory protein 1 alpha (MIP-1α) and 1 beta (MIP-1β), Interferon gamma-induced protein 10 (IP-10), Monokine induced by interferon gamma (MIG), Regulated on Activation, Normal T Cell Expressed and Secreted (RANTES), monocyte chemotactic protein 1 (MCP-1), Epidermal growth factor (EGF), Granulocyte colony-stimulating factor (G-CSF), Fibroblast growth factor (FGF), Hepatocyte growth factor (HGF) and Vascular endothelial growth factor (VEGF) (Table 2).

**Table 2:** Changes in serum cytokines (pg/mL)

	**T1**	**T2**	**T3**	**T4**	**T5**	**ANOVA**
IL-1β	9.2 ± 20	4.7 ± 12	4.5 ± 12	4.3 ± 13	12 ± 21	0.421
IL-2	3.3 ± 8.3	1.5 ± 3.8	1.3 ± 2.5	1.9 ± 4.9	3.6 ± 7.1	0.969
IL-5	9.3 ± 11	9.2 ± 11	12 ± 11	11 ± 11	9.2 ± 11	0.934
IL-7	22 ± 21	16 ± 20	15 ± 18	19 ± 19	27 ± 20	0.500
IL-10	22 ± 53	16 ± 38	15 ± 42	17 ± 39	16 ± 37	0.989
IL-13	7.8 ± 6.2	6.2 ± 5.9	7.8 ± 6.5	7.7 ± 6..4	8.1 ± 7.3	0.711
IL-15	70 ± 29	60 ± 28	62 ± 29	63 ± 26	81 ± 39	0.175
IL-17	0.6 ± 2.5	0.1 ± 0.2	1.0 ± 3.4	0.1 ± 0.1	1.2 ± 3.5	0.446
TNF-α	0.9 ± 1.7	0.6 ± 1.1	0.6 ± 0.7	0.7 ± 0.9	0.8 ± 1.1	0.947
INF-α	43 ± 21	2 9 ± 21	26 ± 24	36 ± 24	44 ± 21	0.182
INF-γ	15 ± 13	15 ± 14	14 ± 13	14 ± 14	20 ± 15	0.904
GM-CSF	106 ± 223	77 ± 182	4 5 ± 115	80 ± 182	86 ± 183	0.918
MIP-1α	50 ± 22	45 ± 20	44 ± 36	47 ± 22	53 ± 17	0.785
MIP-1β	44 ± 20	42 ± 25	53 ± 37	56 ± 27	55 ± 21	0.356
IP-10	243 ± 139	213 ± 119	214 ± 165	187 ± 128	314 ± 272	0.230
MIG	41 ± 27	27 ± 19	28 ± 28	29 ± 21	35 ± 23	0.361
RANTES	3489 ± 2072	2972 ± 2565	3002 ± 3874	3154 ± 2963	4553 ± 3501	0.607
MCP-1	229 ± 55	199 ± 48	293 ± 112	221 ± 84	248 ± 59	0.147
EGF	1.7 ± 2.8	0.9 ± 1.6	2.8 ± 6.3	1.1 ± 1.6	2.4 ± 2.8	0.368
G-CSF	113 ± 59	89 ± 66	92 ± 99	104 ± 64	119 ± 64	0.603
FGF	18 ± 24	13 ± 24	13 ± 16	14 ± 16	18 ± 16	0.764
HGF	89 ± 27	64 ± 23	114 ± 68	156 ± 96	134 ± 36	0.577
VEGF	3.4 ± 4.0	3.2 ± 3.9	4.1 ± 4.0	3.5 ± 4.2	3.7 ± 3.9	0.949

Time points are before induction of anaesthesia (T1), at the end of surgery (T2), at 6 hours after surgery (T3), at the day after surgery (T4), and at 6 days after surgery (T5). Values are mean ± standard deviation.

We found no significant correlations between IL-6 and the patient’s clinical parameters sex (R = 0.049), body mass index (R = 116) or American Society of Anesthesiologists score (R = 116), while there was a significant (p = 0.005) correlation between IL-6 and age (R = - 0.327).

## Discussion

In this study we characterized a large panel of systemic cytokines in order to know if and how much these molecules are regulated in case of a major, but standardized musculoskeletal injury. Our main focus was effects of surgery on these changes, rather than a comparison to a reference or control group, and the preoperative levels therefore were used as references. THA creates a pathophysiological condition that resembles the clinical characteristics of polytrauma, and since bacterial infections were absent at the time of surgery, the immune response was triggered by endogenous stimuli. The major observations were significantly increases in the proinflammatory cytokines IL-6, IL-8 and IL-16 in the postoperative course. These mediators were insignificantly increased at the end of surgery, while IL-1Rα, IL-2R and IL-12 were decreased at this time point. These observations probably reflect hemodilution during major surgery, and our findings support the concept that a major musculoskeletal trauma is associated with a restricted cytokine response in the systemic circulation [6].

It is important to identify inflammatory markers so as to use them as possible targets for the development of new pharmacological approaches in trauma patients. However, traumatically injured patients are exhibiting a host response to pain, and hemorrhage may be superimposed to produce shock, which may enhance the physiological and immunologic responses. It is therefore clear that traumatized and surgical patients cannot be considered similarly under a generic category of inflammation.

The production of cytokines is transient, and since the efficiency is high, their activities have to be tightly controlled. The biological activities of cytokines are modulated by several different, but highly specific strategies, which involve inhibitory cytokines, soluble receptors and receptor antagonists. Then, cytokines may be characterized as either proinflammatory or anti-inflammatory based on their predominant action. However, it should be noted that the common and clear-cut classification of cytokines as either pro or anti-inflammatory might be misleading. The type, duration, and also the extent of cellular activities induced by one particular cytokine can be influenced considerably by the nature of the target cells, the micro-environment of a cell, the type of neighboring cells, cytokine concentrations, the presence of other cytokines, and even on the temporal sequence of several cytokines acting on the same cell. Also the age of the patients may to some extent influence the cytokine response. We found a significant, but poor correlation to age.

The pro-inflammatory cytokines TNF-α and IL-1β have in general been considered to be responsible for the non-hepatic manifestations of the acute-phase response, and to stimulate the release of other cytokines, including IL-6 [7-9]. Our investigation, like others [6,10], questions the use of TNF-α and IL-1β as clinical markers of traumatic inflammation. In contrast, a direct relationship has been confirmed between elevated levels of IL-6 and IL-8 and degree of injury [11-14]. IL-6 is the principal regulator of most acute-phase protein genes and regulates local and systemic inflammatory responses, including the synthesis of hepatic acute-phase reactants like C-reactive protein [15,16]. We found that IL-6 levels were increased at 4 h and at 1 day after surgery. This is in agreement with a previous study in stable trauma patients [17]. Sustained high levels of IL-6 have been associated with increased severity of tissue injury and have correlated with subsequent development of post injury complications [18,19].

The increases in IL-8 were short-lived at 6 hour after surgery and reflect that IL-8 is a chemokine that attracts polymorphonuclear neutrophil cells and macrophages into the wound site [20]. Typically, at 24 h the neutrophil population is at its maximum, and the activity of these neutrophils may play a critical role in recovery. In a study evaluating clinical outcome in children following blunt trauma, serum IL-8 level at admission was identified as the most important determinant of post injury mortality [21] On the other hand, Eotaxin, a chemoattractant cytokine for eosinophiles, was reduced in the postoperative course. These observations probably reflect the interplay by different inflammatory cells following a musculoskeletal injury.

IL-16 is produced by different cell types and acts as an immunomodulatory cytokine, which induces lymphocyte migration, expression of proinflammatory IL-1β, IL6 and TNF-α and modulates apoptosis [22,23]. It is expressed in a variety of pathologic conditions [24], and after trauma plasma IL-16 levels was transiently increased as compared with non-injured controls [25]. High levels immediately after injury suggest that IL-16 may be a component of the acute phase response. However, in those with worse outcome the levels were lower than in less severely injured patients. In vitro experiments have shown that lower concentrations of IL-16 stimulate monocyte secretion of inflammatory cytokines, whereas higher amounts seem to have inhibitory effects [26]. Then, higher levels of IL-16 following injury may suppress the peripheral adaptive immune response, which may protect the injured patient from the consequences of an exaggerated response.

IL-12 is known to be the principal agent that induces naive T-helper cells to assume the Th1 phenotype [27]. Our results are in agreement with previous reports that injury in humans is followed by a diminished capacity to produce IL-12 by peripheral mononuclear cells [28,29]. This may explain at least in part the apparent inability to maintain Th1 function and cytokine production which has been noted in clinical studies approximately 1 week after serious injury. Depressed capacity for IL-12 production has correlated with the development of multiorgan failure [30]. These findings are also in concert with those of a recent report showing diminished IL-12 production in patients in whom sepsis developed after gastrointestinal surgery [31].

In humans, a reciprocal relation between diminished IL-12 production and increased IL-10 production has been shown at approximately 1 week after injury [31]. Traditionally IL-10 has been considered a main antiinflammatory cytokine since septic complication after trauma has been associated with increased levels of this cytokine. In our patients no anti-inflammatory cytokines were increased. On the contrary the anti-inflammatory cytokines IL-1RA were decreased immediately post surgery, but this was probably due to hemodilution. IL-2R was increased at 6 days after surgery. IL-2 is known to enhance dopaminergic transmission and serves as a neuromodulatory molecule [32], and an increase in IL-2R may reflect post injury interplay between this proinflammatory cytokine and its soluble receptor. However, it should be emphasized that the increases in IL-2R were modest, and at 6 days after surgery, the increase may be questioned.

## Conclusions

We conclude that a major, but controlled musculoskeletal trauma is followed by significant increases in the proinflammatory cytokines IL-6, IL-8 and IL-16 and significant decreases in IL-12 and Eotaxin. Otherwise there were modest changes in the systemic cytokine kinetics and no significant expression of anti-inflammatory cytokines.

## Abbreviations

THA: Total hip arthroplasty; ANOVA: Analysis of variance; IL: Interleukin; RA: Receptor alpha; TNF-α: Tumor necrosis factor alpha; INF: Interferon; G-CSF: Granulocyte colony-stimulating factor; GM-CSF: Granulocyte macrophage colony-stimulating factor; MIP-1α: Macrophage inflammatory protein 1 alpha; MIP-1β: Macrophage inflammatory protein 1 beta; IP-10: Interferon gamma-induced protein 10; MIG: Monokine induced by interferon gamma; RANTES: Regulated on Activation, Normal T Cell Expressed and Secreted; MCP-1: Monocyte chemotactic protein 1; EGF: Epidermal growth factor; FGF: Fibroblast growth factor; HGF: Hepatocyte growth factor; VEGF: Vascular endothelial growth factor.

## Competing interests

The authors declare that they have no competing interest.

The study was not financed by any public or private organization.

## Authors’ contributions

OR and PB designed and carried out the study. JR and SPL carried out the analyses. OR drafted the manuscript. All authors have read and approved the final manuscript.
